# NR2B-Tyr phosphorylation regulates synaptic plasticity in central sensitization in a chronic migraine rat model

**DOI:** 10.1186/s10194-018-0935-2

**Published:** 2018-11-06

**Authors:** Xue-Ying Wang, Hui-Ru Zhou, Sha Wang, Chao-Yang Liu, Guang-Cheng Qin, Qing-Qing Fu, Ji-Ying Zhou, Li-Xue Chen

**Affiliations:** 1 Laboratory Research Center, The First Affiliated Hospital of Chongqing Medical University, Chongqing, China 1st You Yi Road, Yu Zhong District, Chongqing, 400016 China; 2 Department of Neurology, The First Affiliated Hospital of Chongqing Medical University, Chongqing, China

**Keywords:** Chronic migraine, Central sensitization, NR2B-Tyr phosphorylation, Synaptic plasticity, TEM, Golgi-cox staining

## Abstract

**Background:**

Although the mechanism of chronic migraine (CM) is unclear, it might be related to central sensitization and neuronal persistent hyperexcitability. The tyrosine phosphorylation of NR2B (NR2B-pTyr) reportedly contributes to the development of central sensitization and persistent pain in the spinal cord. Central sensitization is thought to be associated with an increase in synaptic efficiency, but the mechanism through which NR2B-pTyr regulates synaptic participation in CM-related central sensitization is unknown. In this study, we aim to investigate the role of NR2B-pTyr in regulating synaptic plasticity in CM-related central sensitization.

**Methods:**

Male Sprague-Dawley rats were subjected to seven inflammatory soup (IS) injections to model recurrent trigeminovascular or dural nociceptor activation, which is assumed to occur in patients with CM. We used the von Frey test to detect changes in mechanical withdrawal thresholds, and western blotting and immunofluorescence staining assays were performed to detect the expression of NR2B-pTyr in the trigeminal nucleus caudalis (TNC). NR2B-pTyr was blocked with the Src family kinase inhibitor 4-amino-5-(4-chlorophenyl)-7-(t-butyl)-pyrazolo [3,4-d] pyrimidine (PP2) and the protein tyrosine kinase inhibitor genistein to detected the changes in calcitonin gene-related peptide (CGRP), substance P (SP), and the synaptic proteins postsynaptic density 95 (PSD95), synaptophysin (Syp), synaptotagmin1 (Syt-1). The synaptic ultrastructures were observed by transmission electron microscopy (TEM), and the dendritic architecture of TNC neurons was observed by Golgi-Cox staining.

**Results:**

Statistical analyses revealed that repeated infusions of IS induced mechanical allodynia and significantly increased the expression of NR2B Tyr-1472 phosphorylation (pNR2B-Y1472) and NR2B Tyr-1252 phosphorylation (pNR2B-Y1252) in the TNC. Furthermore, the inhibition of NR2B-pTyr by PP2 and genistein relieved allodynia and reduced the expression of CGRP, SP, PSD95, Syp and Syt-1 and synaptic transmission.

**Conclusions:**

These data indicate that NR2B-pTyr might regulate synaptic plasticity in central sensitization in a CM rat model. The inhibition of NR2B tyrosine phosphorylation has a protective effect on threshold dysfunction and migraine attacks through the regulation of synaptic plasticity in central sensitization.

## Background

Migraine is an incapacitating neurovascular disorder that substantially affects the quality of both life and work of patients. According to the number of headache days suffered per month, migraine can be classified as episodic migraine (EM) or chronic migraine (CM). The prevalence of CM is 1–3% in the general population and 2.5% of migraine patients develop CM each year, which seriously affects their life and work of patients [1, 2]. In addition, the high medical expenses and the decreased ability of CM patients to work place an enormous financial burden on society. The severe headache and high morbidity in CM seriously harm patients’ physical and mental health. Therefore, the World Health Organization (WHO) has listed CM as one of the four most serious chronic dysfunction diseases [3].

The physiopathology of CM is poorly understood. Most research conducted to date has suggest that activation of the trigeminovascular system (TGVS) contributes to the migraine development [4, 5]. Hyperexcitability of neurons lead to neuropathic pain and trigger central sensitization. Central sensitization refers to increased synaptic efficacy in somatosensory neurons in the dorsal horn of the spinal cord following intense peripheral noxious stimuli, tissue injury or nerve damage. This heightened synaptic transmission can lead to a reduction in the pain threshold, the spread of pain sensitivity to non-injured areas and amplification of the pain responses [6]. In chronic migraine, the release of calcitonin gene-related peptide (CGRP) and other excitatory neurotransmitters from the central terminals of trigeminal ganglion (TG) neurons could repetitively excite second-order neurons in the trigeminal nucleus caudalis (TNC), leading to central sensitization and the manifestation of hyperalgesia and allodynia [7, 8]. Therefore, the neuron activation caused by enhanced synaptic transmission in central sensitization may plays a very important role in the maintenance of CM.

Additionally, most studies have suggested that the development and maintenance of central sensitization are largely dependent on the activation of the glutamate N-methyl-D-aspartate (NMDA) receptors [9]. The combination of NMDA receptors and transmitters leads to an intracellular cascade that triggers a series of biochemical reactions resulting in alterations in the structure and function of the synapse, and these synaptic changes greatly enhance excitatory synaptic transmission and thus contribute to chronic pain [10]. Some research on the brain has shown that the N-methyl D-aspartate receptor subtype 2B (NR2B) subunit is the most important tyrosine-phosphorylated protein, and the phosphorylation of NR2B receptor subunits has been proposed to lead to increased Ca2+ entry through the receptor in both central sensitization and NMDA-dependent synaptic plasticity [11, 12]. However, the mechanism through which NR2B participates in CM-related central sensitization by altering synaptic plasticity has not been reported. We conducted a preliminary study and found that NR2B-pTyr might contribute to CM in rats, which manifested as decreased pain thresholds and exaggerated pain responses [13]. Based on our previous studies, NR2B-pTyr was blocked by the administration of PP2 and genistein to investigate synaptic plasticity-related protein expression, the synapse ultrastructure, and the dendritic spine numbers and thus illustrate the resulting changes in the structural plasticity of the synapse. According to our data, NR2B-pTyr participates in the central sensitization-related mechanism of CM by regulating synaptic plasticity.

In this study, we aimed to explore the possible activity-dependent synaptic plasticity of NMDA receptors in CM, and our findings indicated that inhibition of NR2B-pTyr regulation of synaptic plasticity in central sensitization might be a novel and promising candidate for future treatment or prevention of CM.

## Methods

### Animals

Total up to 149 male adult Sprague-Dawley rats (250–300 g) were provided by the Experimental Animal Center of Chongqing Medical University (Chongqing, China). The experimental groups are shown in Table 1. Rats were allowed free access to water and food and were housed at 23 ± 1 °C under a 12/12 h light-dark cycle. Before any experimental procedures, all animals were acclimated for at least 7 days. All the experiments were conducted in accordance with the National Institutes of Health Guide for the Care and Use of Laboratory Animals (NIH Publications No. 80–23, revised 1996). Because this model induces pain in animals, the number of rats used was the minimum necessary to achieve a sufficient level of power for the statistical power.

**Table 1:** Animal numbers in each group

Experimental group	Animals used					Mortality	Total
	VFT	WB	IF	TEM	Golgi-Cox		
sham	10^a^	6	6	6	6		24
CM	10^a^	6	6	6	6	2	26
CM + DMSO	10^a^	6	6	6	6	2	26
CM + PP2 (7.3 nmol)	10					1	11
CM + PP2 (73 nmol)	10^a^	6	6	6	6	1	25
CM + genistein (100 ng/g)	10					1	11
CM + genistein (300 ng/g)	10^a^	6	6	6	6	2	26
Total	20	30	30	30	30	8	149

^a^Indicates shared with other experiments, do not count

### Craniotomy and cannula fixation

Rats were fitted with a cranial chamber, deeply anaesthetized with 10% chloral hydrate (i.p., 0.4 g/kg body weight), and placed in a stereotaxic apparatus (ST-51603; Stoelting Co, Chicago, IL, USA). Following disinfection with iodophor and alcohol, an incision was made along the midline of each rat’s head to fully expose the skull. A skull drill was used to perform a 1-mm-diameter craniotomy in the right frontal bone (+ 1.5 mm from the bregma and + 1.5 mm lateral to the bregma), and a sterile stainless-steel cannula with a plastic cup (RWD, Shenzhen, China) was affixed to the bone using dental cement. The end of the cannula opened onto the dura, allowing inflammatory soup (IS) or phosphate-buffered saline (PBS) to contact the dura. A matched obturator cap was used to seal the cannula. After surgery, antibiotics were topically applied to prevent any infections in the operation region. The rats were then maintained at approximately 37 °C on an electric heating blanket and housed separately until complete recovery from anaesthesia. The rats were allowed recover for at least 7 days prior to dural infusions. All the rat experiments were approved by the Ethics Committee of the Department of First Affiliated Hospital of Chongqing Medical University Medical Research.

### Repeated dura infusions

A CM rat model was established by repeated infusions of IS to the dura in conscious rats. We modeled recurrent trigeminovascular or dural nociceptor activation that is assumed to occur in patients with CM, as described previously [14]. Rats were placed in a box that allowed free movement for the infusion of IS or PBS to the dura. The IS contained 1 mM histamine, 1 mM serotonin, 1 mM bradykinin, and 0.1 mM prostaglandin E2 in PBS (pH 7.4). What is said above chemicals were obtained from Sigma-Aldrich (St. Louis, MO, USA). We provided a steady infusion of 2 μl of IS or PBS was provided through the cannula for 10 min while each rat was freely moving. The tube was left in place for at least 5 min after the infusion to allow the IS or PBS to diffuse into the tissue surrounding the dura, and the cap was returned to the cannula after the infusion. In addition, we inspected the skin and dental cement seal around the cap to ensure no leakage of IS or PBS outside the dura onto the skin. The rats were randomly divided into two groups and infused with IS or PBS for 7 days.

### Animal groups and treatment

Animals were randomly divided into the following groups: the (1) sham group, (2) CM group, (3) CM + dimethyl sulphoxide (DMSO) group, (4) CM + PP2 group, and (5) CM + genistein group. The animals in the sham group were slowly infused with 2 μL of PBS (pH 7.4) into the dura, as described above, whereas those in the CM group were infused with 2 μL of IS. To investigate the role of NR2B-pTyr in the intracellular events after CM, we dissolved the NR2B-pTyr inhibitor PP2 (Abcam, USA) or genistein (Beyotime, China) in DMSO and injected it into the lateral ventricle (− 1.0 mm rear from the rear of the bregma, + 1.5 mm lateral to the bregma, 4.0 mm from the skull plane) with the designated treatment solution (5 μL). An equivalent volume of DMSO was injected into the lateral ventricle as a control. The doses of PP2 (7.3 and 73 nmol) and genistein (100 and 300 ng) used in this study were based on previous studies [13, 15].

### Tactile sensory testing

As previously described, we used the von Frey test to detect the mechanical threshold in the periorbital and hind paw regions. Mechanical thresholds were tested before the first IS or PBS infusion to serve as the baseline. In addition, the test was performed 24 h after each dural infusion and before the next IS or PBS stimulation (*n* = 10, each group). Tactile sensory testing was performed 24 h after the administration of PP2, genistein or DMSO (n = 10, each group) to determine these infusions on mechanical thresholds. Briefly, the rats were placed in the testing apparatus and were acclimated to the testing apparatus during training periods before and after the cannula implantation surgery. Pressure thresholds were determined by applying an electronic von Frey device (Electrovonfrey, model no: 2391, IITC Inc., Woodland Hills, CA, USA), and the assigned force values ranged from 0 to 800 g. According to the manufacturer’s instructions, the pressure probe tip was applied to the periorbital region and hindpaw region of the rats, and the threshold was automatically recorded when the rat quickly retracted its head or hind paw away from the rigid tip. The results for the PBS group were considered to indicate the control mechanical threshold. Threshold values were measured at least three times at each site, with an interval of at least 1 min between tests. The results are presented as the thresholds in g ± standard deviations (SDs). The data were recorded separately for each time point.

### Western blot analysis

We examined the expression of total NR2B (tNR2B), pNR2B-Y1472, pNR2B-Y1252, PSD95, Syp, Syt-1, and CGRP through a western blot assay (*n* = 6 in each group). Twenty-four hours after the administration of PP2 and genistein, the rats were euthanized, and the brains were removed. The TNC tissue was then separated. Cut TNC tissues into pieces and homogenized in radioimmunoprecipitation assay (RIPA) lysis buffer (sc-24,948, Beyotime, China) with protease inhibitor (Beyotime, China) and phosphatase inhibitor (Beyotime, China) at 4 °C for 2 h. The homogenate was centrifuged at 14,000 rpm at 4 °C for 20 min, and the protein concentrations were then determined using a Bicinchoninic Acid (BCA) Protein Assay Kit (Beyotime, China). The supernatant was used as a whole-cell protein extract. Equal amounts of protein were loaded onto a sodium dodecyl sulphate-polyacrylamide gel electrophoresis (SDS-PAGE) gel (Beyotime, China), electrophoresed, and transferred to a polyvinylidene difluoride (PVDF) membrane (Millipore, USA). The membrane was then blocked with 5% nonfat milk at 37 °C for 2 h and incubated with primary antibodies, including anti-NR2B (1:1000, Proteintech), anti-NR2B phospho Y1252 (1:500, Abcam), anti-NR2B phospho Y1472 (1:500, Abcam), anti-PSD95 (1:1000, Abcam), anti-synaptophysin (1:5000, Abcam), anti-synaptotagmin1 (1:500, Bioss), anti-CGRP (1:2000, Abcam), and anti-β-actin (1:5000, Proteintech, USA) at 4 °C overnight. The membranes were washed with Tris-buffered saline Tween 20 (TBST) buffer three times and incubated with a secondary antibody (1:5000, Zhongshan Golden Bridge Bio, China) at 37 °C for 2 h. The immunoblots were probed with a western blot detection kit (Advansta, USA) and visualized with an imaging system (Fusion, Germany). β-actin was used as a loading control to normalise the protein levels.

### Immunofluorescence staining

Rats were anaesthetized and transcardially perfused with 0.9% saline, followed by 4% paraformaldehyde in 0.1 M PBS 1 day after the administration of PP2 or genistein. Regions from the medulla oblongata to the first cervical cord were separated immediately, post-fixed in 4% paraformaldehyde for 24 h, and then sequentially immersed in solutions of sucrose with increasing concentrations (20% to 30%) until the tissue sank to the bottom. Segments of the TNC were cut into 10-μm-thick sections with a cryostat (Leica, Japan). All the sections were stored at − 80 °C for later use. For immunofluorescence staining, the sections were washed three times with PBS and permeabilised with 0.3% Triton X-100 (Beyotime, China) in 0.1 M PBS at 37 °C for 10 min and then incubated with 10% normal goat serum (Boster, China) at 37 °C for 30 min, using a neuronal marker (anti-neuronal nuclei (NeuN), mouse, 1:200, Novus), anti-PSD95 (1:500, rabbit, Abcam), anti-synaptophysin (1:500, rabbit, Abcam), anti-synaptotagmin1 (1:200, rabbit, Bioss), anti-CGRP (1:50, mouse, Santa Cruz Biotechnology, Santa Cruz, CA, USA), and anti-SP (1:50, mouse, Abcam) at 4 °C overnight. Then, after three washes with PBS, the sections were incubated with secondary antibodies Alexa Fluor 488-conjugated goat anti-rabbit immunoglobulin G (IgG, 1:200, Beyotime, China), Alexa Fluor 488-conjugated goat anti-mouse IgG (1:200, Beyotime, China), and Cy3-conjugated goat anti-mouse IgG (1:200, Beyotime, China) at 37 °C for 90 min. Microphotographs were obtained analysed with a fluorescent confocal microscope (ZEISS, Germany). PBS rather than primary antibody was applied to the negative control sections, and no positive signals were detected. The expression levels of CGRP, SP, PSD95, Syp and Syt-1 in the TNC were detected by immunofluorescence staining (*n* = 6 in each group, five images per animal), and five sections from each rat were randomly selected. A 20x objective was used to capture PSD95- and Syt-1-immunoreactive cells and the intensity of Syp immunoreactivity. A 10x objective was used to capture bilateral CGRP and SP immunoreactivity in the TNC, and CGRP and SP expression did not differ between the two sides. The number of positive cells was calculated as the mean of the numbers obtained from five images.

### Transmission electron microscopy

Six rats per group were anaesthetized and perfused with 2.5% glutaraldehyde, and their brains were dissected and removed. The TNC was separated and incubated overnight in 4% glutaraldehyde at 4 °C for 24 h. Then, the TNC was cut into 1-mm^3^ pieces with a blade. Post fixing, embedding, sectioning and staining were performed at Chongqing Medical University. Briefly, the 1-mm^3^ tissue blocks from the TNC were washed three times with PBS and fixed in 1% osmium tetroxide for two hrs. In addition, the tissue blocks were dehydrated in a series of graded aqueous ethanol for 10 min each (50%/70%/90%/2 × 100%). In addition, the tissue blocks were transferred to 100% propylene oxide for 15 min, followed by graded resin infiltration and embedding. Ultrathin sections were prepared on a Leica Ultracut T using a 45-degree diamond histoknife. The tissue was washed two times with distilled water and stained en bloc with 2% uranyl acetate and lead citrate for 45 min. Images were taken using a JEM-1400 PLUS transmission electron microscope (TEM) and analysed using Image Pro Plus. Synaptic morphology parameters were measured at 50000x. The width of the synaptic cleft and the thickness of the postsynaptic density (PSD) were measured using a multi-point averaging method and Guldner’s [16] method. The synaptic interface curvature was obtained using Jones’ [17] method (*n* = 6 in each group, five images per animal).

### Golgi-cox staining

One day after the administration of PP2 or genistein, the rats were injected intraperitoneally with a lethal dose of chloral hydrate to induce anaesthestia (*n* = 6 in each group, five images per animal). The brains were removed as soon as possible without perfusion, and the tissue was rinsed in double-distilled water for 2–3 s to remove blood from the surface. An FD Rapid Golgi Stain Kit™ (FD NeuroTechnologies—Columbia, MD, USA) was used for the tissue preparation and staining procedure. The entire Golgi-Cox staining procedure was conducted in strict accordance with the manufacturer’s user manual and material safety datasheet. The extracted brains were immersed in Rapid Golgi-Cox solution (“Solutions A/B”) for 14 days (the solution was changed once after 24 h) at room temperature (RT) with low ambient light, transferred into cutting solution (“Solution C”), sectioned on a vibratome (Leica VT 1200S, Japan) at 200 μm to ensure that whole (untransected) neuronal arbours could be accommodated and then mounted on gelatine-coated slides. The slides were further developed and processed according to the manufacturer’s instructions and then coverslipped with Permount™ Mounting Medium (Fisher Scientific Co, Waltham, MA, USA). Briefly, the dendrites within the region were imaged with a Zeiss microscope (Axio Imager A2) using 40x and 64x objectives, and the dendritic spines were quantified by an experimenter who was blinded to the group of each sample [18].

### Statistical analysis

The data are expressed as the means ± SDs. Statistical analyses were performed with SPSS 22.0 (SPSS Inc., Chicago, IL, USA), and graphs were generated by GraphPad Prism 7 (GraphPad Software, San Diego, CA, USA). The mechanical thresholds of the sham and CM groups were assessed using two-way analysis of variance (ANOVA) followed by a Bonferroni post hoc test. One-way ANOVA followed by a Bonferroni post hoc test was used to compare the differences among multiple groups. Statistical differences between two groups were analysed using independent-sample t-tests. *P* < 0.05 was considered to indicate statistical significance.

## Results

### The mechanical threshold is reduced after the induction of CM in rats

**Fig. 1:**
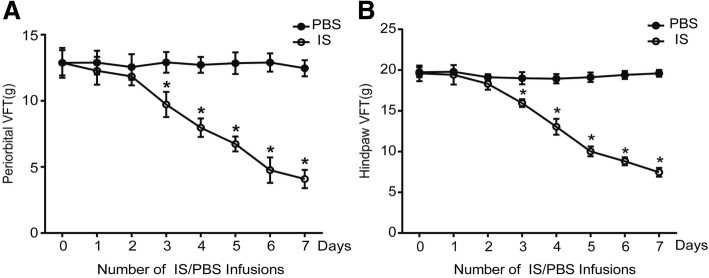
Development of mechanical allodynia in rats. **a** The pain thresholds of the periorbital region were significantly decreased after three days of IS infusions compared with those detected after three days of PBS infusions. **b** A significant decrease in the hindpaw thresholds was observed after three days of IS infusions compared with those detected after three days of PBS infusions (*n* = 10 each group, *P < 0.01)

We used von Frey monofilaments to test the mechanical threshold of the periorbital and hindpaw regions of rats, and the results are shown in Fig. 1. The PBS group was used as a control. Prior to injection, there was no difference in the withdrawal responses to mechanical stimuli in the periorbital (Fig. 1a) or hindpaw (Fig. 1b) region between the IS and PBS groups. The basal pain threshold of the PBS group did not change over time. Compared with the PBS group, the mechanical thresholds of the periorbital and hindpaw regions were significantly reduced in the IS group starting on the third day.

### Changes in total NR2B and phosphorylated NR2B at tyrosine 1472 and 1252 in the TNC after CM in rats

**Fig. 2:**
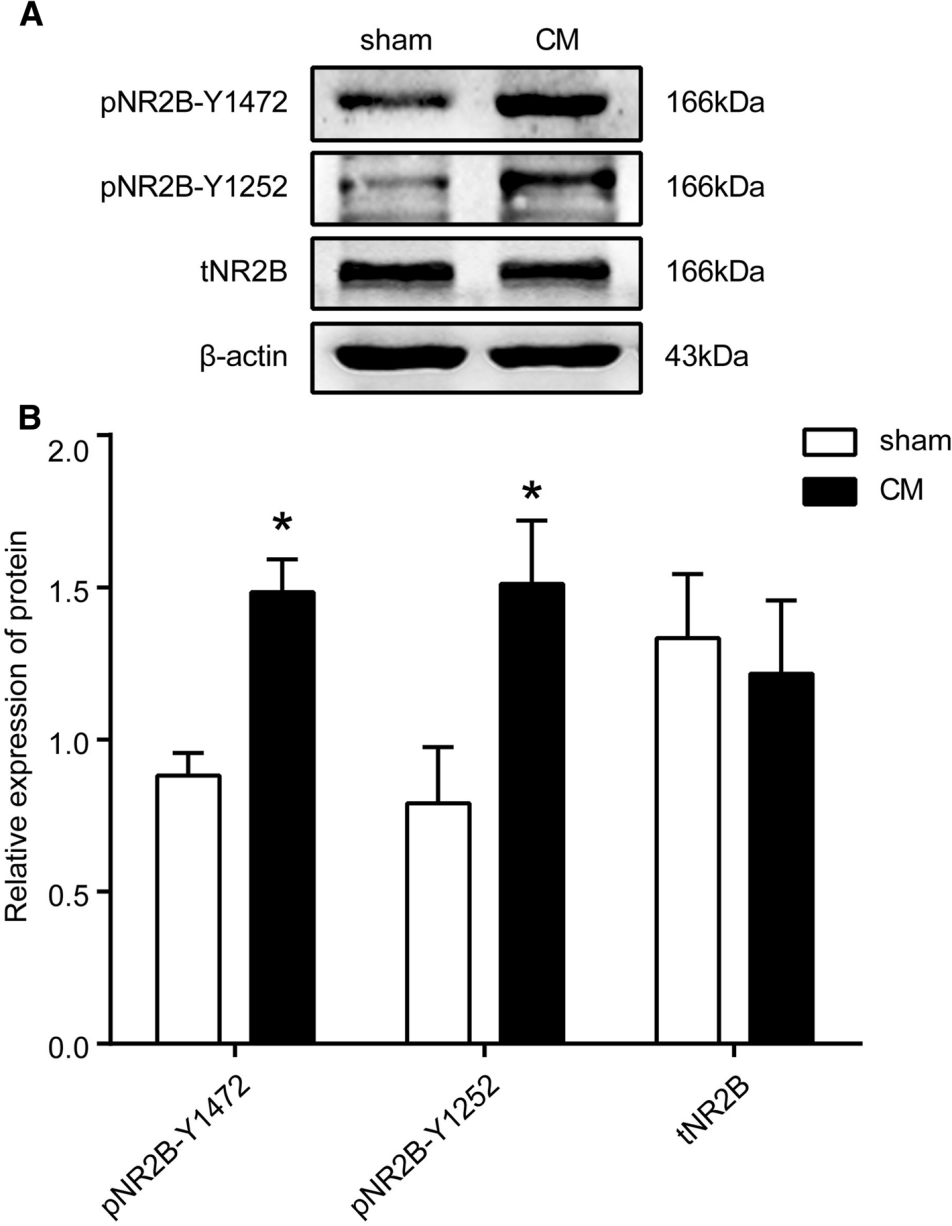
Expression of NR2B and tyrosine-phosphorylated NR2B in the TNC of CM rats. **a** Representative western blots of NR2B-Y1472, NR2B-Y1252 and tNR2B. **b** The phosphorylation of the NR2B receptor subunit at tyrosine 1472 and 1252 was significantly increased in the CM group compared with the sham group. However, the expression of tNR2B in the CM group was indistinguishable from that in the sham group (*n* = 6 in each group, *P < 0.05)

To investigate the changes in NR2B and its phosphorylated forms in CM, we observed the expression of tNR2B and the levels of NR2B-pTyr in CM rats. For these experiments, the rats were killed, their TNC tissues were dissected, and the expression levels of NR2B and NR2B-pTyr were analysed by a western blot analysis. The results revealed that the phosphorylation levels of the NR2B receptor subunit phosphorylated at tyrosine 1472 and 1252 were significantly increased in the CM group compared with those in the sham group (*P* < 0.05). However, the expression of NR2B in the CM group was indistinguishable from that in the sham group (*P* > 0.05) (Fig. 2).

### NR2B-Tyr phosphorylation was associated with allodynia

**Fig. 3:**
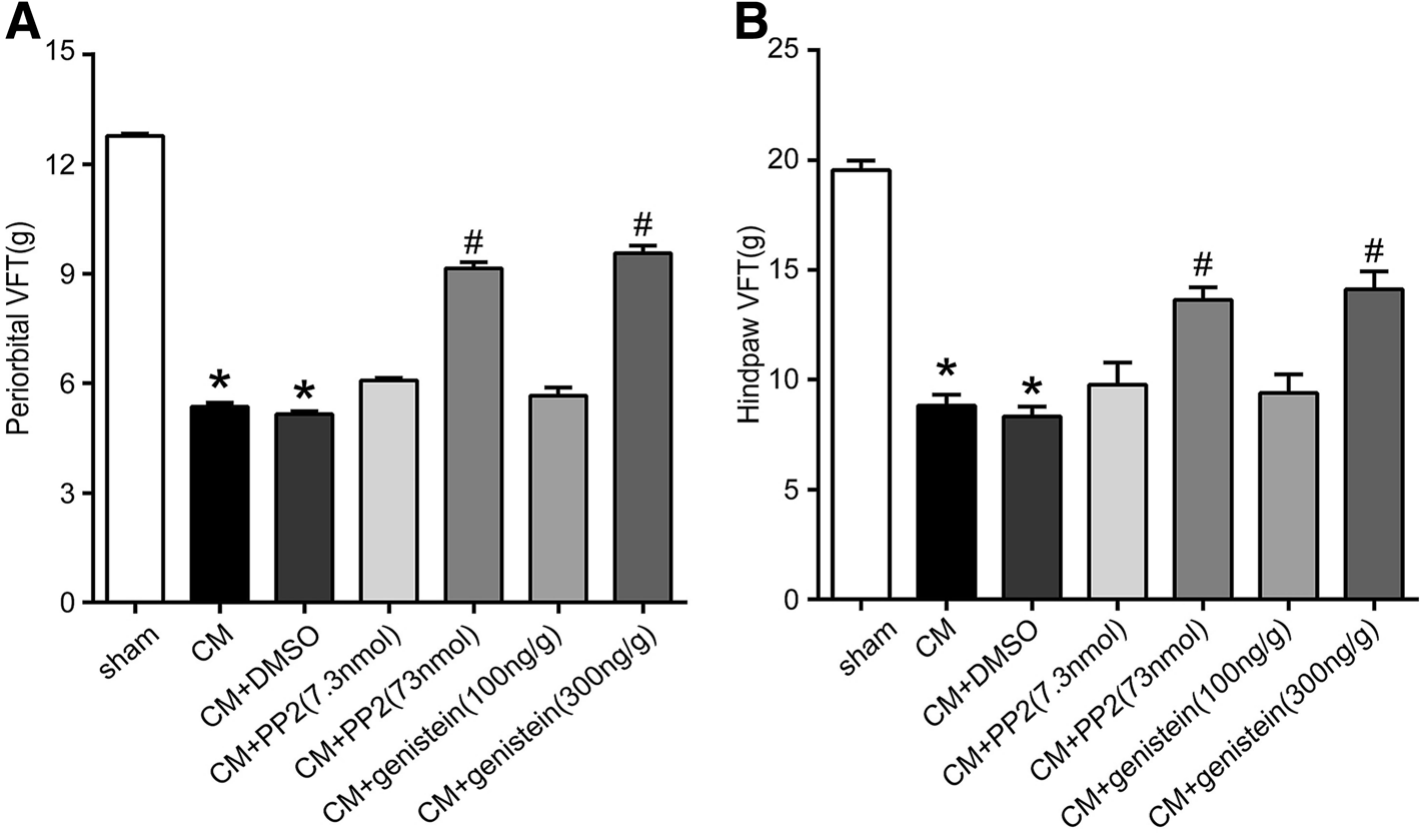
Periorbital pressure and paw withdrawal thresholds after the administration of PP2 and genistein. **a** Periorbital pressure thresholds of the different groups. Compared with the sham group, the CM group showed a significant decrease in the periorbital pressure thresholds, but no significant difference in the von Frey test was found between the CM and CM + DMSO groups. Compared with that found for the CM + DMSO group, the administration of PP2 and genistein significantly increased the periorbital pressure thresholds. There was no significant difference in the von Frey test results among the CM + PP2 (7.3 nmol), genistein (100 ng/g) and CM + DMSO groups. The pain thresholds of the periorbital region obtained in the CM + PP2 (73 nmol) and genistein (300 ng/g) groups were significantly increased compared with those in the CM + DMSO group. **b** Paw withdrawal thresholds of the different groups. Compared with the sham group, the CM group showed a significant decrease in the paw withdrawal thresholds, but there was no significant difference in the von Frey test results between the CM and CM + DMSO groups. Compared with that found for the CM + DMSO group, the administration of PP2 and genistein significantly increased the periorbital pressure thresholds. There was no significant difference in the von Frey test results among the CM + PP2 (7.3 nmol), genistein (100 ng/g) and CM + DMSO groups, but there were significant differences in the von Frey test results mong the CM + PP2 (73 nmol), genistein (300 ng/g) and CM + DMSO groups (*n* = 10, each group, *P < 0.01 compared with the sham group, ^#^*P* < 0.01 compared with the CM + DMSO group)

The von Frey monofilament-based approach was used to determine whether NR2B-pTyr is related to allodynia in CM rats. As shown in Fig. 3a and b, the periorbital pressure thresholds and paw withdrawal thresholds to mechanical stimulation decreased after CM. However, there was no significant difference in the hind paw pain thresholds and between the CM and CM + DMSO groups. The administration of PP2 and genistein significantly improved periorbital pressure thresholds and paw withdrawal thresholds. No significant differences in the periorbital pressure thresholds or paw withdrawal thresholds were detected among the CM + PP2 (7.3 nmol), genistein (100 ng/g) and CM + DMSO groups, but high doses of PP2 (73 nmol) and genistein (300 ng/g) exerted a significant protective effect against allodynia. We then performed western blot and immunofluorescence staining analyses of the high-dose (PP2/genistein) group.

### Changes in NR2B and phosphorylated NR2B at tyrosine 1472 and 1252 in the TNC after PP2 and genistein administration

**Fig. 4:**
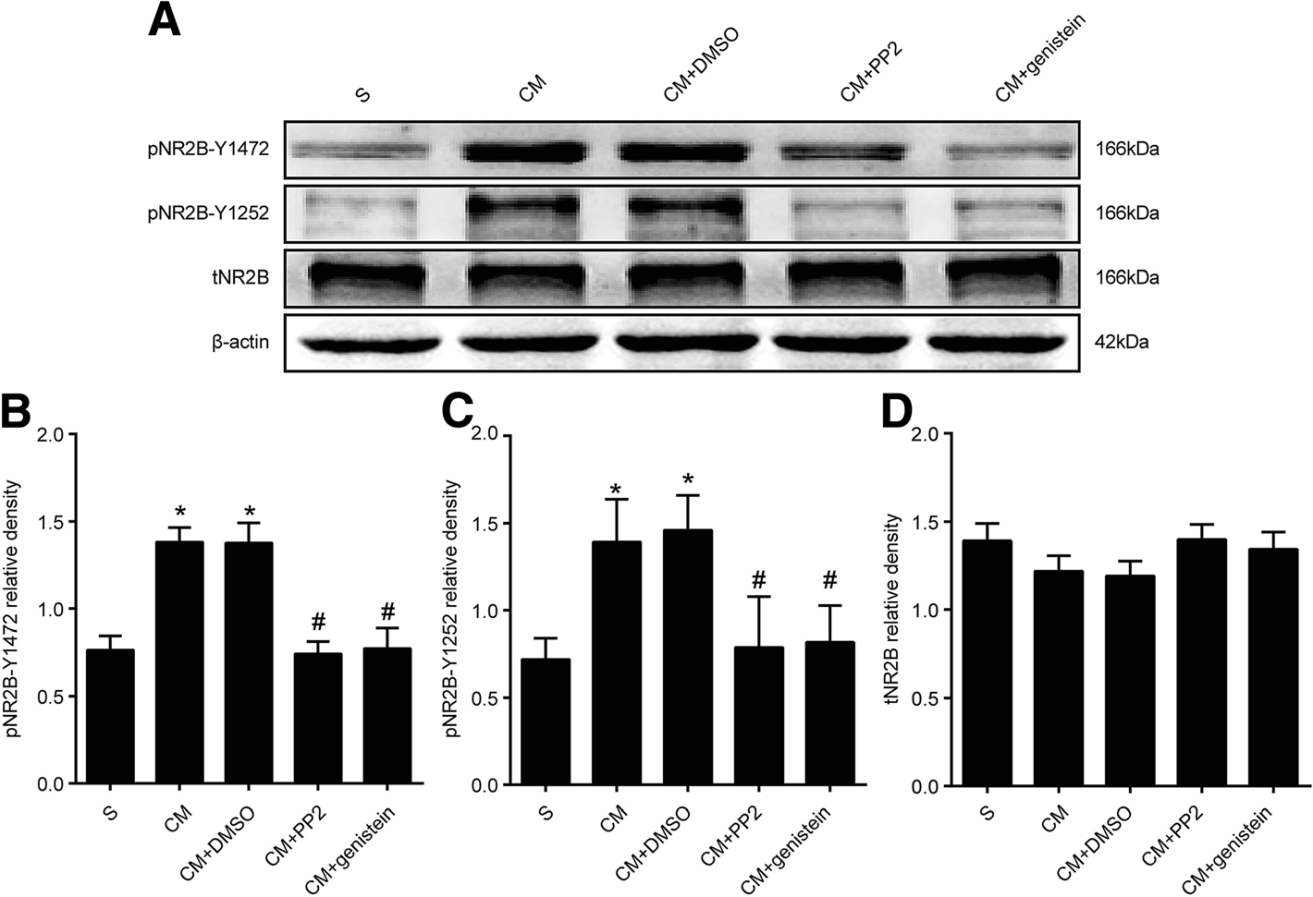
Expression of phosphorylated NR2B (pNR2B-Y1472 and pNR2B-Y1252) and tNR2B in each group. **a** The four rows show tNR2B, pNR2B-Y1472, pNR2B-Y1252 and β-actin. **b** The protein levels of pNR2B-Y1472 in the TNC were higher in the CM group than in the sham group. Compared with that found in the CM + DMSO group, PP2 and genistein significantly decreased the levels of pNR2B-Y1472. **c** The protein levels of pNR2B-Y1252 in the TNC were higher in the CM group than in the sham group. Compared with that found for the CM + DMSO group, PP2 and genistein significantly decreased the level of pNR2B-Y1252. **d** There was no significant difference in the expression of tNR2B among the different groups (*n* = 6 in each group, *P < 0.05 compared with the sham group, ^#^*P* < 0.05 compared with the CM + DMSO group)

To investigate the changes in NR2B and its phosphorylated forms after the administration of PP2 and genistein, we measured the tNR2B and tyrosine-phosphorylated NR2B (pNR2B-Y1472 and pNR2B-Y1252) expression levels in CM rats 1 day after the administration of PP2 and genistein by western blotting (Fig. 4a). The level of pNR2B-Y1472 was significantly higher in the CM group than in the sham group. However, our data did not show a significant difference in the phosphorylation level of this subunit between the CM and CM + DMSO groups, which suggests that intracerebroventricular injections of DMSO (control group) did not change the level of pNR2B-Y1472. PP2 and genistein significantly decreased the phosphorylation level of pNR2B-Y1472 (Fig. 4b), and the same result was also observed with pNR2B-Y1252 (Fig. 4c). However, we did not observe any significant differences in the expression of tNR2B among the groups (Fig. 4d).

### NR2B-pTyr was associated with CGRP and SP expression

**Fig. 5:**
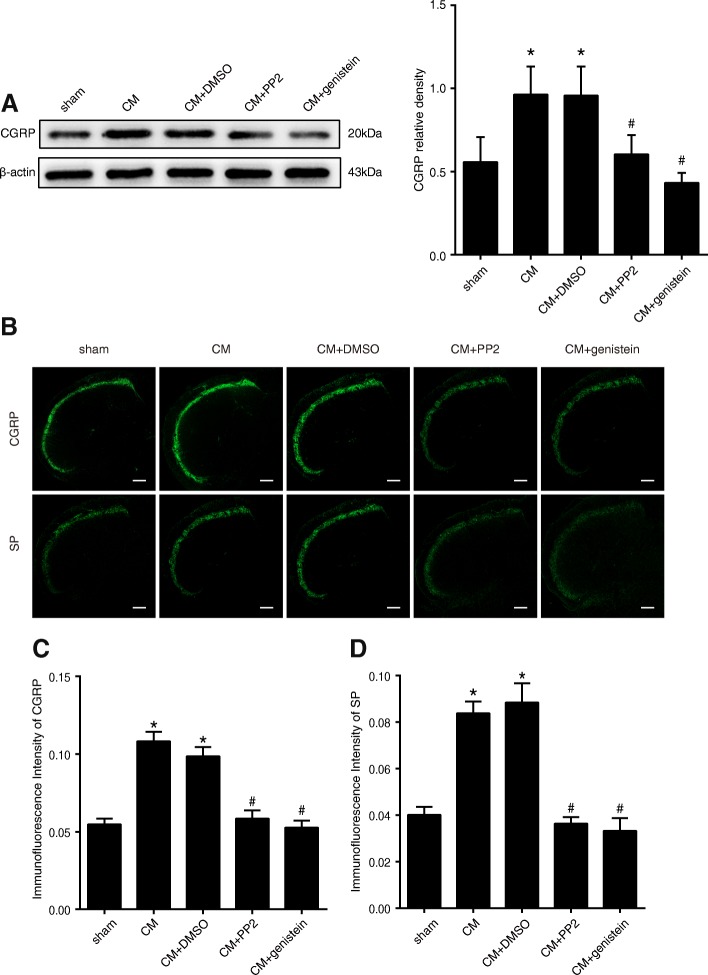
Expression levels of CGRP in the TNC and immunofluorescence staining for CGRP and SP in the TNC. **a** The expression of CGRP was significantly increased in the CM group compared with that in the sham group, and no significant difference in the expression of CGRP was found between the CM and CM + DMSO groups. The expression of CGRP in CM rats was significantly decreased after the administration of PP2 and genistein (*n* = 6 in each group, five images per animal, *P < 0.05 compared with the sham group, #P < 0.05 compared with the CM + DMSO group). **b** The bilateral immunoreactivity of CGRP and SP in the TNC was investigated by immunofluorescence staining (the image shows only one side because no difference was found between the two sides). **c** The fluorescence intensities of CGRP in the TNC were elevated in the rats belonging to the CM groups compared with those in the sham group, and no significant difference in the expression of CGRP was found between the CM and CM + DMSO groups. PP2 and genistein treatment decreased the CGRP fluorescence intensities. **d** The fluorescence intensities of SP in the TNC were elevated in the rats of the CM groups with those in the sham group, and there was no significant difference in the expression of SP between the CM and CM + DMSO groups. PP2 and genistein treatment decreased the SP fluorescence intensities (*n* = 6 in each group, *P < 0.05 compared with the sham group, ^#^*P* < 0.05 compared with the CM + DMSO group, scale bar = 200 μm)

CGRP and SP are important in migraine pathophysiology, and the synthesis and release of CGRP and SP by primary afferent neurons are very important for the induction of central sensitization following peripheral injury, as well as the maintenance of central sensitization in inflammatory pain [19, 20]. A western blot analysis showed that the CGRP levels were significantly increased in the CM group compared with those in the sham group. However, our data did not show a significant difference in CGRP expression between the CM and CM + DMSO groups, suggesting that intracerebroventricular injections of DMSO (control group) did not change CGRP expression in the TNC. The inhibition of NR2B-Tyr phosphorylation by PP2 and genistein reduced the levels of CGRP (Fig. 5a). CGRP and SP immunoreactivity in the TNC was then evaluated by immunofluorescence staining (Fig. 5b). The fluorescence intensity of CGRP was higher in the CM group than in the sham group, and PP2 and genistein treatment weakened the elevation of CGRP immunoreactivity induced by IS infusions, which was consistent with the western blotting results (Fig. 5c). The same result was also obtained for SP (Fig. 5d).

### Tyrosine phosphorylation of NR2B was involved in the mechanism of chronic migraine through synaptic plasticity

#### The level of NR2B-pTyr regulated the expression of the synapse-associated proteins PSD95, Syp and Syt-1

**Fig. 6:**
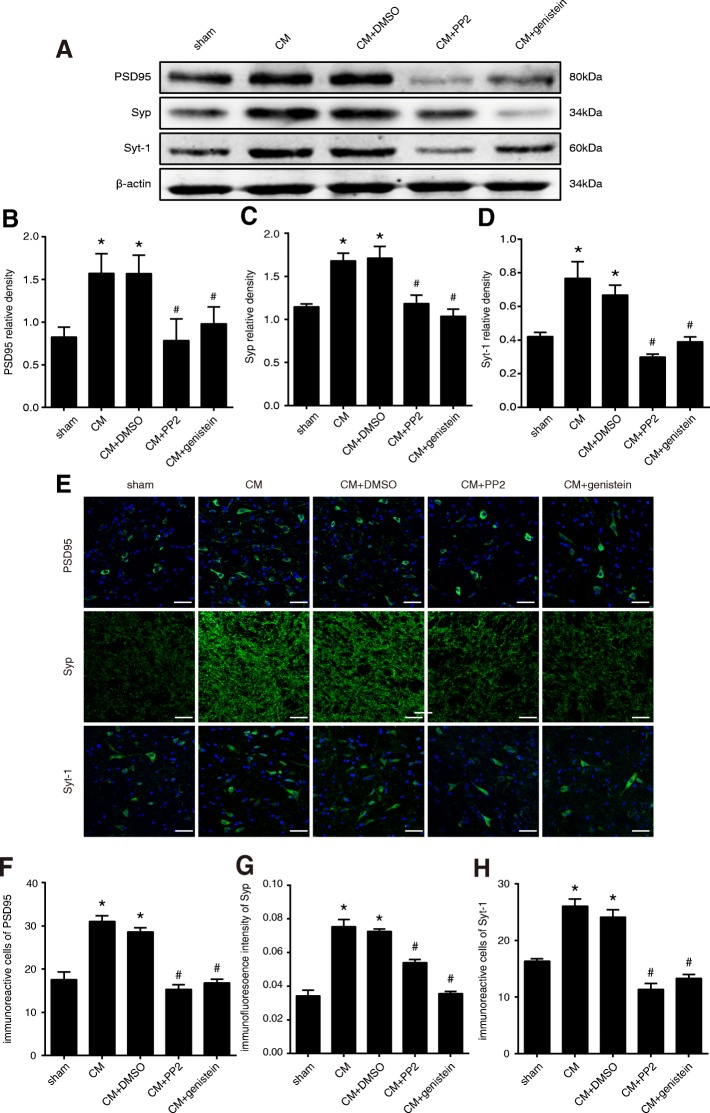
Expression and immunofluorescence staining of PSD95, Syp, and Syt-1 in the TNC in each of the five groups. **a** The expression levels of PSD95 (**b**), Syp (**c**), and Syt-1 (**d**) were significantly increased in the CM group compared with those in the sham group, and no significant difference in the expression of PSD95 was found between the CM and CM + DMSO groups. The expression of PSD95 in CM rats significantly decreased following the administration of PP2 and genistein (*n* = 6 in each group, five images per animal, *P < 0.05 compared with the sham group, #P < 0.05 compared with the CM + DMSO group). **e** Immunofluorescence staining for PSD95, Syp, and Syt-1 in the TNC. Semiquantitative analyses showed that the numbers of PSD95 (**f**) and Syt-1 (**h**)-positive cells were significantly increased in the CM group compared with those in the sham group; moreover, PP2 and genistein significantly reduced the numbers of PSD95- and Syt-1-positive cells. Semiquantitative analyses showed that the fluorescence intensity of Syp (**g**) in the TNC was higher in the rats belonging to the CM group than in those of the sham group, and there was no significant difference in the expression of Syp between the CM and CM + DMSO groups. PP2 and genistein treatment decreased the Syp fluorescence intensity (*n* = 6 in each group, *P < 0.05 compared with the sham group, ^#^*P* < 0.05 compared with the CM + DMSO group, scale bar = 50 μm)

To test whether NR2B-pTyr affects synaptic plasticity in CM rats, we first measured the expression of the synapse-associated proteins PSD95, Syp, and Syt-1 (Fig. 6a). A western blot analysis showed that the expression levels of PSD95 (Fig. 6b), Syp (Fig. 6c) and Syt-1 (Fig. 6d) were significantly increased in the CM group compared with those in the sham group, and no significant difference was found between the CM and CM + DMSO groups. In addition, PP2 and genistein significantly decreased the expression levels of PSD95, Syp, and Syt-1. The immunoreactivity of PSD95, Syp and Syt-1 in the TNC was then evaluated by immunofluorescence staining (Fig. 6e). The fluorescence intensity of Syp (Fig. 6g) and the numbers of PSD95 (Fig. 6f) and Syt-1 (Fig. 6h)-positive cells were elevated in the CM group compared with those in the sham group, but there were no obvious differences between the CM + DMSO and CM groups. PP2 and genistein treatment weakened the elevation of Syp immunoreactivity induced by IS infusions and reduced the numbers of PSD95- and Syt-1-immunoreactive cells. These findings were consistent with the results of the western blot analysis.

#### The level of NR2B-pTyr regulated the synaptic ultrastructure observed by TEM

**Fig. 7:**
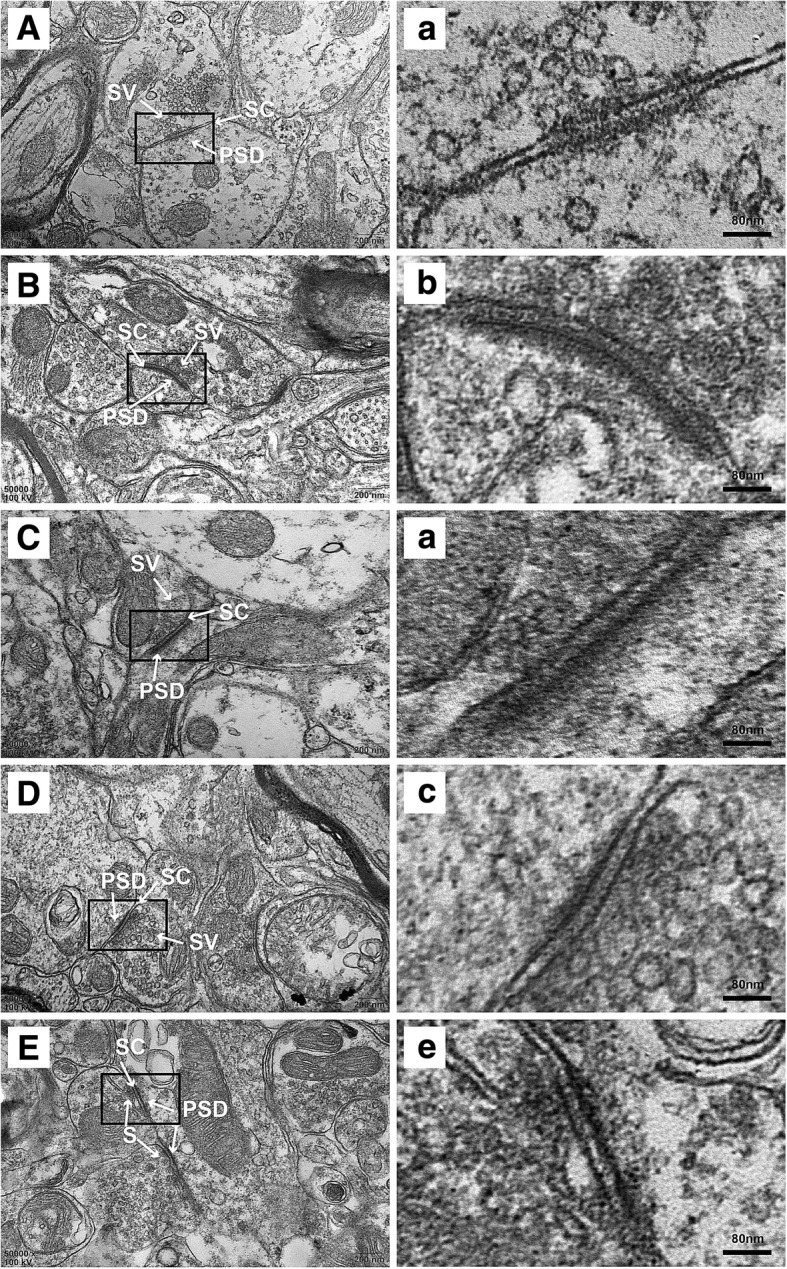
Synaptic structure of the TNC neurons in each group. **a**, *a*: sham group; **b**, *b*: CM group; **c**, *c*: CM + DMSO group; **d**, *d*: CM + PP2 group; **e**, *e*: CM + genistein group. PSD, postsynaptic density; SC, synaptic cleft; SV, synaptic vesicle. *a*-*d* show enlarged versions of the images in **a**-**d**. Scale bars = 200 nm (**a**-**d**). Scale bars = 40 nm (*a*-*d*)

The synaptic structure of TNC neurons in each group was observed under a TEM, and representative images are shown in Fig. 7.

In the sham control group, the presynaptic and postsynaptic membranes were clear and had complete outlines, the synaptic cleft was clear, and abundant PSD was detected. Compared with the sham control group, the CM group exhibited vague presynaptic membranes, augmented synaptic clefts, thicker postsynaptic densities, longer active zones, and an increased synaptic interface curvature. The administration of PP2 and genistein restored the related synapse morphological indicators (Table 2).

**Table 2:** Parameters of the synaptic interface in each of the five groups in the TNC of CM rats

*n* = 6	Sham	CM	CM + DMSO	CM + PP2	CM + genistein
Thickness of the PSD/nm	22.24 ± 1.2321	40.51 ± 1.9948*	39.64 ± 1.5399*	25.34 ± 0.9563^#^	30.16 ± 0.6379^#^
Width of the synaptic cleft/nm	19.21 ± 0.8048	32.39 ± 0.4477*	32.37 ± 0.4968*	21.56 ± 0.9589^#^	22.63 ± 1.1490^#^
Active zones/nm	289.42 ± 8.31	529.8396 ± 18.6*	538.99 ± 23.15*	382.52 ± 23.51^#^	360.37 ± 22.64^#^
Synaptic interface curvature	1.080 ± 0.0202	1.331 ± 0.0284*	1.3115 ± 0.0184*	1.1643 ± 0.0156^#^	1.1357 ± 0.0125^#^

n, the number of rats. Data are presented as the means ± SDs. *P < 0.05 compared with the sham control group. ^#^*P* < 0.05 compared with the CM + DMSO control group

#### The level of NR2B-pTyr regulated the number of dendritic spines in TNC neurons

**Fig. 8:**
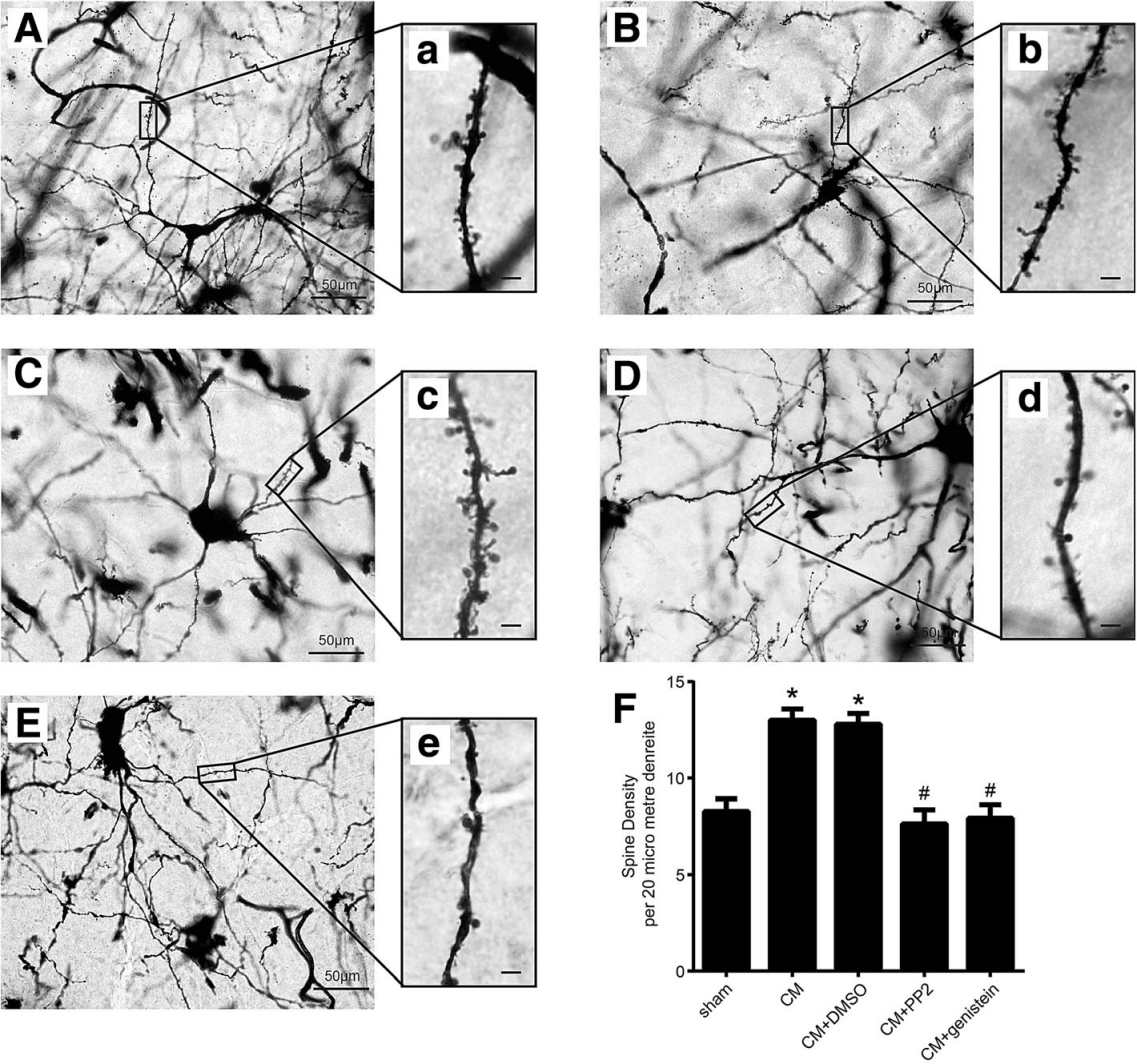
Dendritic spine density of the TNC neurons in each group. **a-e** Low magnification of Golgi-Cox staining in the five groups (**a**: sham group; **b**: CM group; **c**: CM + DMSO group; **d**: CM + PP2 group; **e**: CM + genistein group, scale bar = 50 μm). *a*-*e* Enlarged images of neurons with dendrites that were used for the quantification of the spine density in the five groups; these images correspond to those shown in **a**-**e** (*a*: sham group; *b*: CM group; *c*: CM + DMSO group; *d*: CM + PP2 group; *e*: CM + genistein group, scale bar = 4 μm). **f** Golgi-Cox staining showed that the dendritic spine density was significantly higher in the CM group compared with that in the sham group, and there was no significant difference between the CM and CM + DMSO groups. PP2 and genistein significantly reduced the dendritic spine density, indicating that tyrosine-phosphorylated NR2B reduces the dendritic spine density in CM rats (n = 6 each group, *P < 0.05 compared with the sham group, ^#^*P* < 0.05 compared with the CM + DMSO group)

We performed Golgi-Cox staining to quantify the number of dendritic spines in the five groups (Fig. 8). All neurons selected from each group for the analysis fulfilled the following criteria: (1) the dendrites showed dark and consistent Golgi-Cox staining across their entire length, (2) the dendrites were visibly intact, and (3) the neurons had sufficient space between them to prevent interference during the analysis [21]. A significant increase in the dendritic spine density in the TNC was observed in the rats of the CM group compared with that of the rats belonging to the sham group, and no significant differences were found between the CM and CM + DMSO groups. The administration of PP2 and genistein significantly decreased the dendritic spine density. These data imply that the dendritic spine density was increased in CM rats and was associated with NR2B-pTyr.

## Discussion

In the present study, we used the von Frey test to detect hyperalgesia and allodynia and thus investigate the role of NR2B and NR2B-pTyr in rats with CM induced by repeated infusions of IS. In addition, the use of PP2/genistein to suppress the tyrosine phosphorylation of NR2B ameliorated the hyperalgesia induced by repeated IS stimulation and downregulated the expression of CGRP and SP. Moreover, the inhibition of NR2B-pTyr by PP2/genistein downregulated the expression of the synapse-associated proteins PSD95, Syp, and Syt-1 and even altered the synaptic ultrastructure and the number of dendritic spines to reduce synaptic plasticity. Based on these results, we provide the first evidence showing that NR2B-pTyr in the TNC is involved in the mechanism of CM. The effect of NR2B-pTry was found to be largely associated with its regulation of synaptic plasticity. Therefore, the tyrosine phosphorylation of NR2B and synaptic plasticity might represent potential therapeutic targets in CM.

Central sensitization is a crucial process underlying increased neuronal excitability, and some published evidence indicates that central sensitization plays a role in the maintenance of prolonged migraine pain and might also contribute to migraine chronicity [6, 22, 23]. Repeated dural nociceptor activation specifically leads to gradual worsening of cutaneous hypersensitivity, general neuronal hyperexcitability, persistent cephalic cutaneous hypersensitivity and trigeminal central sensitization [24]. In the present study, we used a CM rat model established by repeated IS stimulation of the dura to repeatedly activate the assumed nociceptors, We discovered that the periorbital and hind paw pain thresholds gradually declined with repeated infusions of IS. This finding suggests that IS induces cephalic and extracephalic allodynia.

NMDA receptors constitute one of the principal types of ionotropic glutamate receptors that mediate fast excitatory synaptic transmission in the central nervous system (CNS). Abundant evidence indicates that NMDA receptors play critical roles in a range of physiological and pathological processes in the CNS. One of the key mechanisms for regulating NMDA receptor function involves the tyrosine phosphorylation of NR2B subunits by the tyrosine kinase Fyn [25, 26]. NR2B contains three tyrosine phosphorylation sites (Y1252, Y1336 and Y1472), and several studies have suggested that pNR2B-Y1472 plays a significant role in the trafficking of NMDA receptors [25, 27, 28]. In an animal model of inflammatory pain, the development of inflammation and hyperalgesia was found to be associated with a rapid and prolonged increase in the pNR2B-Y1472 level. In addition, the inflammation-induced increase in NR2B-pTyr was abolished by genistein, a tyrosine kinase inhibitor, and PP2, a Src family protein tyrosine kinase inhibitor [29]. In agreement with previous studies, we found that pNR2B-Y1472 was involved in IS-induced hyperalgesia. The protein levels of pNR2B-Y1472 were overexpressed in the TNC of CM rats, and only high doses of PP2 (73 nmol) and genistein (300 ng) relieved mechanical hyperalgesia. In addition, we found the same changes in the phosphorylation of Y1252 sites. However, other remaining phosphorylation sites should be further investigated. These results suggest that the phosphorylation of NR2B at Y1472 and Y1252 regulated CM activation in TNC neurons.

CGRP and SP are important in migraine pathophysiology, expressed in trigeminal ganglia neurons and involved in trigeminovascular innervation, and modulation of nociceptive transmission. Additionally, these proteins are used as biological markers of neuronal activation and central sensitization [20, 30]. As shown in previous studies, the plasma CGRP and SP levels are increased during a migraine attack [31, 32]. In our study, upregulated expression levels of CGRP and SP were observed in the TNC of CM rats, and CGRP and SP immunoreactivity was mainly detected in the outer laminae of the TNC, which is likely associated with the processing of nociceptive information. This finding is in line with the results of a previous study [30]. In addition, PP2 and genistein downregulated the levels of CGRP and SP in the TNC, which indicates that NR2B phosphorylation might play a prominent role in neuronal activation and central sensitization in the CM model.

In addition, NR2B-pTyr was reported contributes to the development of persistent pain in the spinal cord by regulate synaptic transmission. Therefore, It’s very interesting to explore the mechanism of NR2B-pTyr contributes to central sensitization in CM. As is well known, excitatory neurotransmission in somatosensory nociceptive pathways is predominantly mediated by glutamatergic synapses [33]. Recent studies have consistently demonstrated that glutamatergic synapses play an important role in sensory transmission, including pain and itch transmission, and contribute to nociceptive sensitization [34, 35]. Many studies have reported that regulation of synaptic plasticity by NR2B-pTyr may play a role in nociceptive transmission in the chronic visceral pain model and neuropathic pain model [36, 37]. Similar to a previous report, we detected many synaptic-related indicators in the CM model to emphasize that its association with the synaptic plasticity of NMDA receptors. PSD95 is preferentially localized to dendritic spines and plays a critical role in the regulation of the size and shape of dendritic spines [38, 39]. Syp, a major integral membrane protein of presynaptic vesicles, is required for vesicle formation and exocytosis and is widely used as a marker for synaptic activity [40]. Syt-1, which acts as the major Ca2 + −sensor for fast presynaptic vesicle exocytosis, is a marker of synaptic transmission. Similar to previous studies, our study revealed that the expression levels of synaptic function/structure-related proteins PSD95, Syp and Syt-1 were increased in CM rats and that PP2 and genistein downregulated these changes. This finding suggests that the expression of synaptic function/structure-related proteins in the TNC is directly related to CM rats and positively correlated with the level of NR2B-pTyr. However, electrophysiological experiments are necessary to more fully describe these changes in synaptic plasticity changes.

## Conclusion

**Fig. 9:**
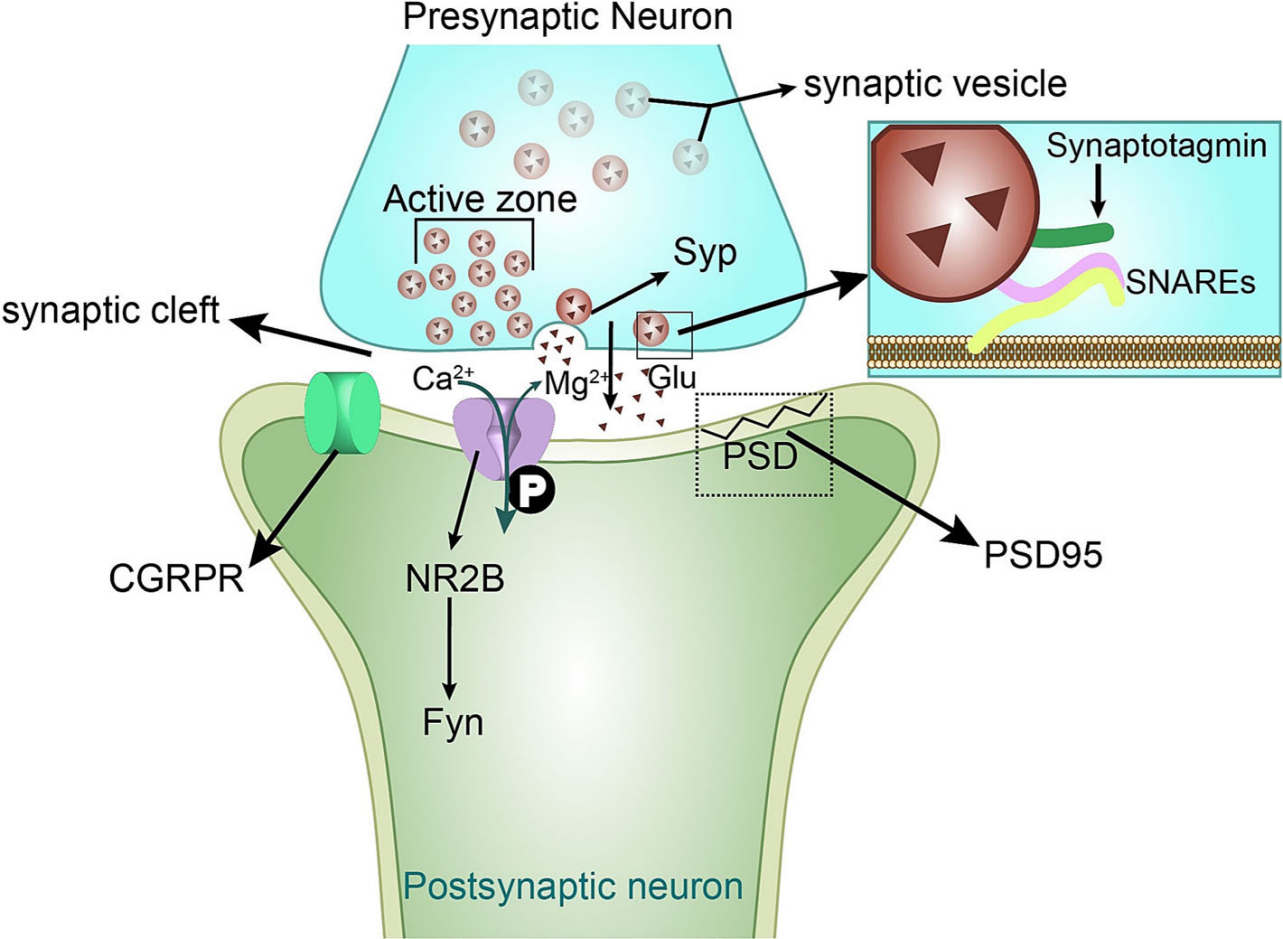
Schematic diagrams of the NR2B-Tyr phosphorylation-mediated regulation of synaptic plasticity in central sensitization

In conclusion, our results demonstrate that NR2B-pTyr contributes to the central sensitization of CM in rats. The inhibition of NR2B tyrosine phosphorylation exerts a protective effect on threshold dysfunction and migraine attacks through the regulation of synaptic plasticity in central sensitization (Fig. 9). This study provides a new perspective on the function of NR2B-pTyr in CM and identifies NR2B as a novel candidate for the treatment of CM in patients.

## Abbreviations

CGRP: Calcitonin gene-related peptide
CM: Chronic migraine
CNS: Central nervous system
DMSO: Dimethyl sulfoxide
IS: Inflammatory soup
NMDA: N-methyl-D-aspartate
NR2B-pTyr: Tyrosine-phosphorylated NR2B
PP2: Phosphorylation 4-amino-5-(4-chlorophenyl)-7-(t-butyl)-pyrazolo[3,4-d] pyrimidine
PSD: Postsynaptic density
PSD95: Postsynaptic density 95
SP: Substance P
Syp: Synaptophysin
Syt-1: Synaptotagmin1
TEM: Transmission electron microscope
TG: Trigeminal ganglion
TGVS: Trigeminovascular system
TNC: Trigeminal nucleus caudalis
